# Neue Auflage der Paris-Klassifikation 2022: Was ist neu?

**DOI:** 10.1007/s00292-024-01340-7

**Published:** 2024-07-03

**Authors:** Tatjana Vlajnic, Lukas Bubendorf

**Affiliations:** Pathologie, Institut für medizinische Genetik und Pathologie, Universitätsspital Basel, Schönbeinstr. 40, 4031 Basel, Schweiz

**Keywords:** Urinzytologie, Harntraktzytologie, Harntrakt, Urotheliale Neoplasien, Oberer Harntrakt, Urine cytology, Urinary tract cytology, Urinary tract, Urothelial neoplasms, Upper urinary tract

## Abstract

Als ein international anerkanntes Befundungssystem hat die Paris-Klassifikation einen globalen Durchbruch in der Standardisierung der Diagnosen in der Urinzytologie erzielt. Basierend auf Erfahrungen der letzten Jahre seit der Erstveröffentlichung werden in der Neuauflage die diagnostischen Kriterien präzisiert und differentialdiagnostische Schwierigkeiten ausführlicher diskutiert. Während der Nachweis eines high-grade Urothelkarzinoms nach wie vor im Vordergrund steht, werden auch weitere Aspekte der Urinzytologie, u. a. die Zytologie des oberen Harntrakts, und die damit verbundenen Herausforderungen thematisiert. Neu werden die low-grade urothelialen Neoplasien nicht mehr als eigenständige Kategorie aufgeführt, sondern in die Kategorie „negativ für high-grade Urothelkarzinom“ (NGHUC) eingeordnet. Die Paris-Klassifikation ist eine wichtige Grundlage für die Abschätzung des Malignitätsrisikos und das weitere klinische Vorgehen.

## Abstract (en)

As an internationally accepted diagnostic system, the Paris classification has achieved a global breakthrough in the standardization of diagnoses in urine cytology. Based on experience over the past few years since its first publication, the new edition of the Paris classification refines the diagnostic criteria and discusses diagnostic pitfalls. While the detection of high-grade urothelial carcinoma remains the main focus, other aspects of urine cytology, including cytology of the upper urinary tract and the associated challenges, have also been addressed. Low-grade urothelial neoplasia is no longer listed as a separate category but is now included in the category “negative for high-grade urothelial carcinoma” (NGHUC). Essentially, the Paris classification provides an important basis for estimating the risk of malignancy and further clinical management.

Mit der erfolgreichen Einführung der Paris-Klassifikation (The Paris System, TPS) in die Routinediagnostik im Jahre 2016 wurde ein international standardisiertes Befundungssystem für die Urinzytologie etabliert [10]. Die Einteilung zytologischer Befunde in diagnostische Kategorien anhand reproduzierbarer morphologischer Kriterien hat bei Zytologen und Urologen weltweit eine breite Akzeptanz gefunden und die Aussagekraft der Urinzytologie deutlich aufgewertet.

Seit der Veröffentlichung der ersten Auflage der Paris-Klassifikation wurde der Stellenwert der Urinzytologie vor und nach der TPS-Ära in zahlreichen Studien untersucht und verglichen. Basierend darauf und in einer online durchgeführten Umfrage mit 450 weltweit tätigen Zytologen [4] wurden einige Aspekte identifiziert, auf die in der initialen Version unzureichend eingegangen wurde, was zu einer überarbeiteten Neuauflage der Paris-Klassifikation geführt hat [13]. Dazu gehören unter anderem die Einteilung plattenepithelialer Atypien, die Problematik degenerativ veränderter Zellen und die Interpretation der Zytologie des oberen Harntrakts (OHT), der nun ein ganzes neues Kapitel gewidmet ist. Zudem wurden verschiedene histologische Varianten des high-grade Urothelkarzinoms (HGUC) berücksichtigt und nukleäre Hypochromasie als seltene Eigenschaft von HGUC-Zellen diagnostisch anerkannt (Abb. 1). Auffallend hypochromatische Zellkerne sind in bis zu 15 % aller HGUC zu finden [6, 9]. Die Ursache und biologische Bedeutung dieser Veränderung ist bisher unbekannt.

**Abb. 1:**
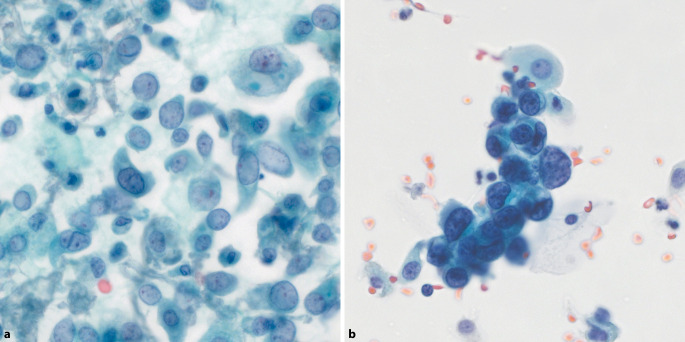
High-grade Urothelkarzinom (HGUC). **a** HGUC mit *hyp*ochromatischen Kernen und hoher Kern-Plasma-Relation. **b** HGUC mit klassischerweise *hyper*chromatischen Kernen

Im Zuge der Überarbeitung wurden die diagnostischen Kriterien für die Paris-Kategorien präzisiert und die Kategorie „low-grade urotheliale Neoplasie“ (LGUN) abgeschafft. Die wichtigsten morphologischen Kriterien für den diagnostischen Algorithmus wie Kern-Plasma-Relation, Kerneigenschaften und Anzahl atypischer Zellen sind in einem Entscheidungsbaum graphisch dargestellt (Abb. 2). Diagnostische Fallstricke in den jeweiligen Kategorien wurden ausführlicher diskutiert und mit zahlreichen illustrativen Beispielen ergänzt. Die diagnostischen Kategorien sind in Tab. 1 zusammengefasst.

**Abb. 2:**
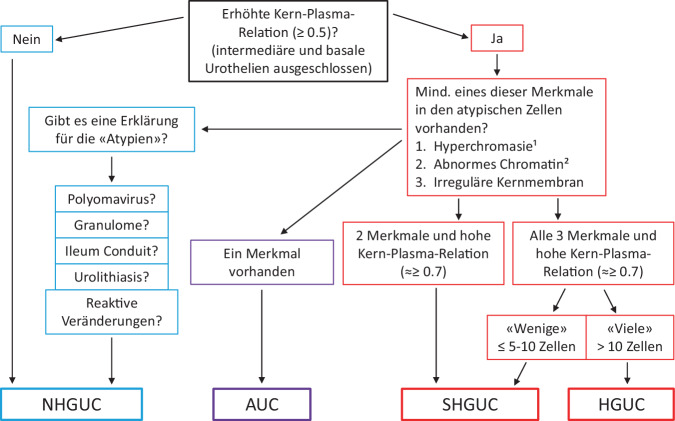
Grafischer Algorithmus des Entscheidungsbaums der Paris-Klassifikation. (Mod. nach Wojcik et al. [14]) ^1^ In einigen Fällen auch Hypochromasie; ^2^ Vergröbertes und unregelmässig verteiltes oder verklumptes Chromatin

**Tab. 1:** Diagnostische Kategorien der Paris-Klassifikation 2022

Nichtdiagnostisch	
*NHGUC*	Negativ für high-grade Urothelkarzinom^a^
*AUC*	Atypische urotheliale Zellen
*SHGUC*	Verdächtig auf high-grade Urothelkarzinom
*HGUC*	High-grade Urothelkarzinom
Andere	Nichturotheliale maligne Neoplasien und andere seltene Veränderungen

^a^Einschließlich low-grade urothelialer Neoplasien

## Kategorie „negativ für high-grade Urothelkarzinom“

**Abb. 3:**
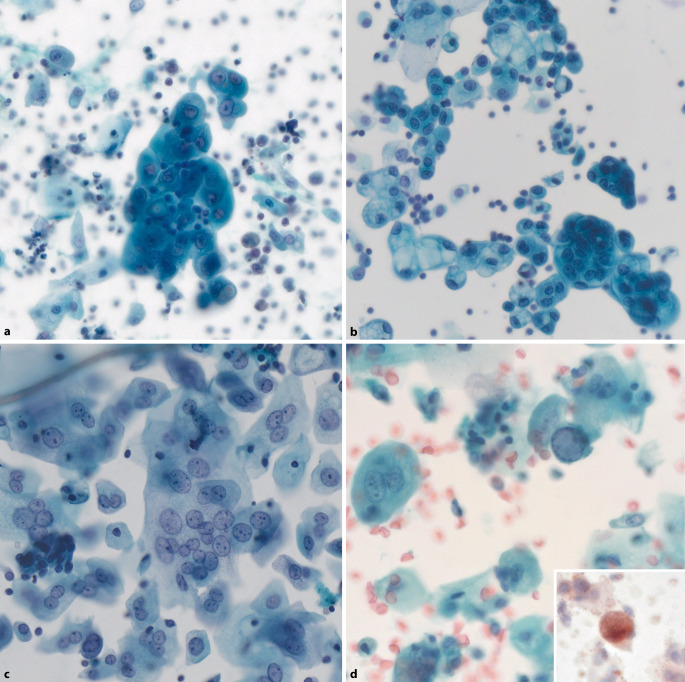
Negativ für high-grade Urothelkarzinom (*NHGUC*). **a** Reaktiv veränderte Urothelien nach intravesikaler BCG(Bacillus Calmette Guérin)-Therapie mit prominenten Nukleolen. **b** Reaktive Veränderungen nach BCG-Therapie mit vakuolisiertem Zytoplasma. **c** Reaktive Veränderungen bei Reizblase mit aktivierten, teils mehrkernigen Deckzellen. **d** Decoy-Zelle (Polyomavirus-Infektion) bei einem Patienten mit Vorgeschichte eines high-grade Urothelkarzinoms vor 5 Jahren: milchglasartiger, viraler Kerneinschluss mit marginalisiertem Kernchromatin. Hohe Kern-Plasma-Relation. *Inset*: Immunzytochemische Positivität mit dem Pan-Polyomavirus-Antiköper SV40

Die Urinzytologie ist ein zentraler Bestandteil der Diagnostik von Erkrankungen der Harnwege sowie in der Nachsorge von Patienten mit bekannten urothelialen Neoplasien. Das Verständnis der unterschiedlichen zugrundeliegenden Pathogenesen von high-grade und low-grade urothelialen Neoplasien, welche in einem Kapitel zusammengefasst wurden, ermöglicht es, den Stellenwert der Paris-Klassifikation besser einzuordnen und sie differenziert einzusetzen. Auch in der überarbeiteten Version liegt der Schwerpunkt im zytologischen Nachweis des potenziell lebensbedrohlichen HGUC. Somit ist die Hauptaufgabe der Paris-Klassifikation die Ermittlung der *Risikoabschätzung*, welche als Entscheidungshilfe für das klinische Management von Patienten dienen soll (Tab. 2). Die Kategorie „negativ für high-grade Urothelkarzinom“ (NHGUC) umfasst somit alle Veränderungen, die nicht mit einem erhöhten Malignitätsrisiko einhergehen. Folglich ist der Begriff „negativ“ bzw. NHGUC nicht mit „kein Nachweis einer Neoplasie“ gleichzusetzen, sondern bedeutet „kein signifikant erhöhtes Risiko für die Entwicklung eines HGUC“. Entscheidend ist das Erkennen einer spezifischen Ursache (z. B. Urolithiasis, Katheter, stattgehabte [BCG-]Therapie, Polyomavirus-Infektion) für *reaktive Veränderungen* (Abb. 3), um den Befund korrekterweise als NHGUC einzuordnen und dem Impuls zu widerstehen, die Kategorie „atypische urotheliale Zellen“ (AUC) zu verwenden. Für die korrekte Interpretation solcher Proben ist die Kenntnis und Korrelation der klinischen und zystoskopischen Befunde unbedingt erforderlich. Den Urologen kommt somit in der integrativen Diagnostik eine bedeutende Rolle zu. Daher sollten sie darüber aufgeklärt werden, dass die Angaben zu

1. Vorgeschichte eines UC,
2. stattgehabten Therapien und
3. zytoskopischen Befunden

für die korrekte Interpretation der zytologischen Befunde unerlässlich sind.

**Tab. 2:** Häufigkeit und relatives Risiko der Paris-Kategorien. (Nach Woycik et al. [13])

Kategorie	Häufigkeit (%)	Malignitätsrisiko^a^ (%)
*Ungeeignet*	0–5	0–16
*NHGUC*	70–90	8–24
*AUC*	5–15	24–53
*SHGUC*	0,5–3	59–94
*HGUC*	0,1–3	76–100

^a^Definiert als Risiko für high-grade Malignität

*AUC* atypische urotheliale Zellen, *NHGUC* negativ für high-grade Urothelkarzinom, *SHGUC *verdächtig auf high-grade Urothelkarzinom, *HGUC* high-grade Urothelkarzinom

**Abb. 4:**
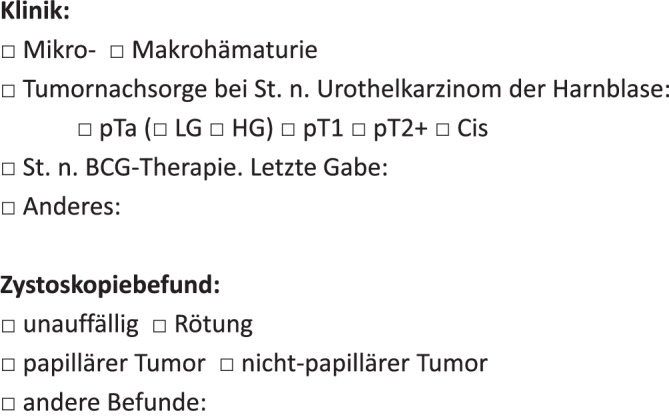
Notwendige klinische Informationen für die Befundung der Urinzytologie. Die Angaben können vordefiniert in die Einsendeformulare eingebaut werden

Um eine standardmäßige Übermittlung dieser Informationen zu gewährleisten, können die notwendigen Angaben vordefiniert zur Auswahl auf den Einsendeformularen für die Urinzytologie integriert werden (Abb. 4). Die Kategorie AUC ist strenggenommen lediglich für Urothelien mit zytologischen Atypien vorgesehen, deren Ausmaß reaktive Veränderungen übersteigt und die aus einem HGUC stammen könnten. Studien haben gezeigt, dass die restriktive Anwendung der AUC-Kategorie eine deutliche Reduktion und eine bessere Eingrenzung der bisher uneinheitlich genutzten Diagnose von „Atypien“ ergeben hat, womit eine weitere Zielsetzung der Paris-Klassifikation erfüllt ist (zusammengefasst in [13]).

**Abb. 5:**
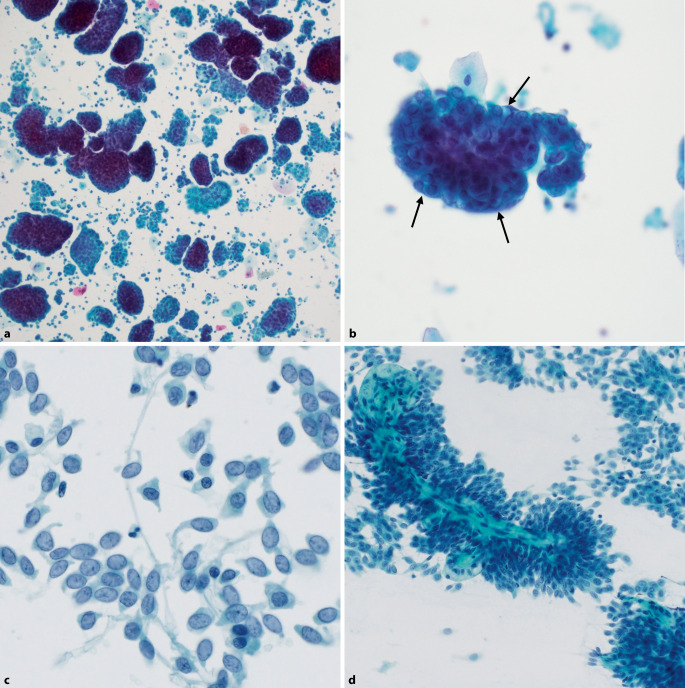
Negativ für high-grade Urothelkarzinom (*NHGUC*). **a,** **b** Knospenartige Verbände von Urothelien mit Deckzellen (*Pfeile*). **c** Monotones Zellbild aus Urothelien mit leichten Kernatypien ohne Hyperchromasie, verdächtig auf low-grade urotheliale Neoplasie. **d** Echte Papillen mit fibrovaskulärem Stiel, vereinbar mit low-grade papillärer urothelialer Neoplasie

Der Stellenwert der Zytologie in der Abklärung *low-grade (papillärer) urothelialer Neoplasien* bleibt auch nach Einführung der Paris-Klassifikation umstritten. Die Kategorie LGUN ist in der neuen Auflage daher nicht mehr enthalten. Wenn eine low-grade (papilläre) Neoplasie zytologisch vermutet wird, wird der Befund definitiv als NHGUC klassifiziert und eine Beschreibung zytologischer Merkmale (monotones Zellbild aus Urothelien mit leichten Kernatypien ohne Hyperchromasie, erhöhte Kern-Plasma-Relation, ggf. mit Nachweis von Papillen; Abb. 5c, d) entweder im Diagnosetext oder im Kommentar vermerkt (Tab. 3). Gründe für die nach wie vor niedrige Sensitivität der Zytologie für eine affirmative Diagnose sind einerseits, dass eine zytologische Unterscheidung von Zellen einer low-grade Läsion und normalen Urothelien sehr schwierig sein kann, und andererseits, dass der Nachweis von echten Papillen – insbesondere in Zytospinpräparaten – nur selten gelingt [5, 15]. Zudem weisen low-grade (papilläre) urotheliale Neoplasien in der Regel kein erhöhtes Progressions- bzw. Malignitätsrisiko auf. An dieser Stelle wird betont, dass der Nachweis von Urothelien mit *geringen Atypien* nicht dazu verleiten soll, den Befund der AUC-Kategorie zuzuordnen.

**Tab. 3:** Diagnosebeispiele

Harnblase (Spülflüssigkeit): Zahlreiche geringgradig atypische Urothelien, teilweise in papillären Verbänden, vereinbar mit low-grade urothelialer Neoplasie *Paris-Klassifikation: Negativ für high-grade Urothelkarzinom*
Harnblase (Spülflüssigkeit): Einige atypische verhornende plattenepitheliale Zellen *Paris-Klassifikation: Anderes – Atypische plattenepitheliale Zellen* Kommentar: Der Nachweis atypischer verhornender Plattenepithelien könnte auf ein (teils) plattenepithelial differenziertes Karzinom hinweisen
Spontanurin: Zahlreiche teils degenerativ veränderte Urothelien ohne wesentliche Kernatypien *Paris-Klassifikation: Negativ für high-grade Urothelkarzinom*
Harnblase (Spülflüssigkeit): Vereinzelt degenerativ veränderte Urothelien mit nicht sicher klassifizierbaren Kernatypien sowie reichlich zytologisch unverdächtige Urothelien. *Paris-Klassifikation: Atypische urotheliale Zellen* Kommentar: Ein high-grade Urothelkarzinom lässt sich nicht ausschließen. Je nach klinischem Korrelat empfehlen wir eine zeitnahe Kontrolle
Nierenbecken rechts (Spülflüssigkeit): Monotones Zellbild aus zahlreichen geringgradig atypischen Urothelien *Paris-Klassifikation: Negativ für „high-grade“ Urothelkarzinom* Kommentar: Der Befund ist verdächtig auf eine low-grade urotheliale Neoplasie und ließe sich, je nach klinischem Korrelat, mittels Fluoreszenz-in-situ-Hybridisierung weiter abklären

## Degenerative Veränderungen

Degenerative Veränderungen urothelialer Zellen sind unspezifisch und können sowohl bei malignen als auch benignen Veränderungen vorkommen. Somit rechtfertigt der alleinige Nachweis degenerativ veränderter Urothelien nicht die Diagnose AUC. Naturgemäß finden sich degenerative Veränderungen häufiger im Spontanurin und in Urinproben aus dem Ileum-Conduit oder der Neoblase als in den Spülflüssigkeiten der Harnblase. Soweit es möglich ist, sollten auch die degenerativ veränderten Zellen nach den gleichen diagnostischen Kriterien beurteilt werden. Für die Interpretation sind auch hier vor allem die *Kerngröße und die Kern-Plasma-Relation* maßgebend. Degenerativ veränderte normale Urothelien behalten in der Regel eine Kern-Plasma-Relation von < 0,5. Ein morphologischer Vergleich mit angrenzenden vitalen Zellen ist häufig hilfreich. Bei Proben mit degenerativ veränderten HGUC-Zellen finden sich meist auch einige vitale Zellen mit vergrößerten Kernen und erhöhter Kern-Plasma-Relation. Sofern eine morphologische Interpretation nicht gelingt und die degenerierten Zellen in ausreichender Anzahl vorhanden sind, kann der Befund mittels eines molekularen Zusatztests (z. B. Fluoreszenz-in-situ-Hybridisierung, FISH) hinsichtlich Dignität weiter abgeklärt werden. Wenn eine definitive Interpretation nicht möglich ist, sollten intermediäre Kategorien (AUC oder „verdächtig auf HGUC“ [SHGUC]) für die Diagnose gewählt werden. Von einer affirmativen HGUC-Diagnose an ausschließlich degenerativ veränderten Urothelien wird abgeraten.

## Atypische plattenepitheliale Zellen

**Abb. 6:**
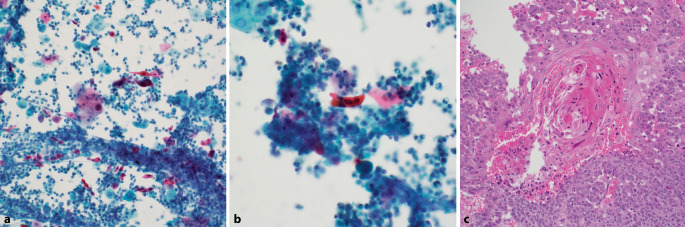
Atypische plattenepitheliale Zellen (Kategorie „Anderes“). **a,** **b** Wenige atypische verhornende Plattenepithelien vor granulozytär-entzündlichem Hintergrund. **c** Histologische Diagnose eines invasiven Plattenepithelkarzinoms desselben Falles

Der Nachweis hochgradig atypischer oder eindeutig maligner plattenepithelialer Zellen in der Urinzytologie umfasst differentialdiagnostisch primäre und sekundäre Plattenepithelkarzinome sowie HGUC mit plattenepithelialer Differenzierung [2, 3, 8]. Atypische plattenepitheliale Zellen sind charakterisiert durch verhorntes/orangeophiles Zytoplasma, vergrößerte und hyperchromatische Kerne mit irregulärer Kernmembran und polygonale oder bizarre Zellformen („Kaulquappen“). In der Regel finden sich bei invasiven Plattenepithelkarzinomen zahlreiche Tumorzellen. In einigen Fällen sind jedoch nur wenige atypische plattenepitheliale Zellen vor einem granulozytär-entzündlichen oder nekrotischen Hintergrund vorhanden (Abb. 6). In Abhängigkeit der Menge atypischer Plattenepithelien und des Ausmaßes zytologischer Atypien sollte der Befund als „*Anderes: Atypische plattenepitheliale Zellen*“ oder „*Anderes: Atypische plattenepitheliale Zellen, verdächtig auf ein Plattenepithelkarzinom oder HGUC* *mit plattenepithelialer Differenzierung*“ kategorisiert werden. Die Kategorie AUC sollte hierfür nicht angewendet werden, da die auffälligen Zellen nicht urothelialer Natur sind. Beim ausschließlichen Nachweis von malignen Plattenepithelien ohne eindeutige maligne Urothelzellen kann es sich sowohl um ein Urothelkarzinom mit überwiegender plattenepithelialer Differenzierung oder um ein reines Plattenepithelkarzinom handeln. Idealerweise sollte eine solche zytologische Diagnose („Anderes“) in einem Kommentar näher erläutert und eine Korrelation mit klinischen Befunden und ggf. eine histologische Abklärung empfohlen werden.

## Zytologie des oberen Harntrakts

**Abb. 7:**
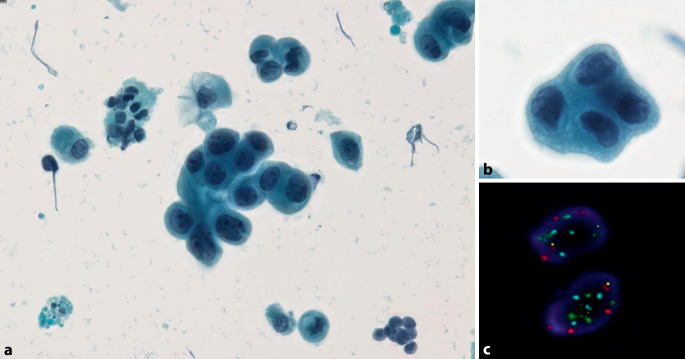
Fluoreszenz-in-situ-Hybridisierung (*FISH*) mit 4 Sonden. **a** Atypische urotheliale Zellen (*AUC*) in der Spülflüssigkeit des Nierenbeckens: Einzeln und in kleinen Verbänden liegende Urothelien ohne wesentlich erhöhte Kern-Plasma-Relation (< 0,7) und mit relativ runden Kernen und diskreten Kernmembranirregularitäten. **b** Zellen desselben Falles bei starker Vergrößerung. **c** FISH-positives Resultat: unregelmäßige Polysomie für die Chromosomen 3, 7 und 17 mit relativem Verlust von 9p21. FISH-Sonden/Fluoreszenzfarben (UroVysion™, Abbott Laboratories, Abbott Park, IL, USA): Chromosom 3 (*rot*), 7 (*grün*), 17 (*aqua*) und 9p21 (*gold*). Als positives FISH-Resultat gilt der Nachweis von unbalancierten numerischen chromosomalen Aberrationen in mindestens 2 Chromosomen und/oder ein kompletter oder relativer Verlust von 9p21 (entsprechend einer homozygoten oder heterozygoten 9p21-Deletion)

Urothelkarzinome des OHT (i.e. Nierenbecken und Ureter) machen lediglich 5–10 % aller Urothelkarzinome aus, sind jedoch mit einer eher ungünstigen Prognose assoziiert [1, 11]. Generell werden für die zytologische Beurteilung der Spülflüssigkeiten aus dem OHT die gleichen morphologischen Kriterien angewendet wie bei der Harnblase. Die Zellen eines HGUC im OHT können jedoch *kleinere Kerne und eine höhere Kern-Plasma-Relation* aufweisen und somit leicht übersehen werden [7]. Wegen erheblicher klinischer Konsequenzen (z. B. Nephrektomie) sollte die Diagnose eines HGUC im OHT vor einer definitiven Therapie nach Möglichkeit histologisch gesichert werden. Sofern dies aufgrund anatomischer Gegebenheiten technisch nicht machbar ist, sollte eine unabhängige Zweitbeurteilung zur Diagnosesicherung eingeholt werden. Die Verdachtsdiagnose einer low-grade urothelialen Neoplasie ist im OHT mit äußerster Vorsicht und Zurückhaltung zu stellen. Nicht selten finden sich in der Spülflüssigkeit dreidimensionale *benigne reaktive urotheliale Verbände *(Abb. 3a, b), die wegen zytomorphologischer Überlappung leicht mit Zellverbänden einer low-grade urothelialen Neoplasie verwechselt werden können. Letztere können im Gegensatz zu benignen Zellverbänden fibrovaskuläre Stränge (Pseudopapillen oder echte Papillen) ausbilden. Zur weiteren Abklärung von unklaren zytologischen Befunden gilt FISH zum Nachweis chromosomaler Aberrationen als eine der am besten etablierten Zusatzmethoden ([12]; Abb. 7).

## Fazit für die Praxis

- Die erhöhte Kern-Plasma-Relation (≥ 0,7) gilt nach wie vor als Kernkriterium für die Diagnose eines high-grade Urothelkarzinoms (HGUC). Dabei ist es wichtig, nichtneoplastische Zellen mit erhöhter Kern-Plasma-Relation (z. B. basale Urothelien, Polyomavirus-infizierte Zellen) anhand ihrer jeweils charakteristischen Merkmale korrekt als solche zu identifizieren.
- Low-grade urotheliale Neoplasien werden neu in der Kategorie „negativ für high-grade Urothelkarzinom“ (NHGUC) aufgeführt, da sie kein signifikant erhöhtes Malignitätsrisiko aufweisen.
- Jeglicher Nachweis atypischer verhornender Plattenepithelien ist ein starker Hinweis auf ein plattenepithelial differenziertes Karzinom und sollte in der Kategorie „Anderes: Atypische plattenepitheliale Zellen“ berichtet werden.
- Aufgrund weitreichender Konsequenzen bei der Diagnose eines HGUC im oberen Harntrakt wird eine histologische Sicherung oder eine Zweitbeurteilung empfohlen.
- Degenerative Veränderungen sind unspezifisch und kommen bei benignen und malignen Läsionen vor. Ihre Interpretation sollte im Kontext der klinischen Information und der übrigen zytologischen Befunde erfolgen.

## References

[1] Commander CW, Johnson DC, Raynor MC et al (2017) Detection of Upper Tract Urothelial Malignancies by Computed Tomography Urography in Patients Referred for Hematuria at a Large Tertiary Referral Center. Urology 102:31–37 28088432 10.1016/j.urology.2016.10.055

[2] Hattori M, Nishimura Y, Toyonaga M et al (2012) Cytological significance of abnormal squamous cells in urinary cytology. Diagn Cytopathol 40:798–803 21309015 10.1002/dc.21645

[3] Khaled H (2013) Schistosomiasis and cancer in egypt: review. J Adv Res 4:461–466 25685453 10.1016/j.jare.2013.06.007 PMC4293882

[4] Kurtycz DFI, Wojcik EM, Rosenthal DL (2023) Perceptions of Paris: an international survey in preparation for The Paris System for Reporting Urinary Cytology 2.0 (TPS 2.0). J Am Soc Cytopathol 12:66–74 36274039 10.1016/j.jasc.2022.09.002

[5] Mccroskey Z, Kliethermes S, Bahar B et al (2015) Is a consistent cytologic diagnosis of low-grade urothelial carcinoma in instrumented urinary tract cytologic specimens possible? A comparison between cytomorphologic features of low-grade urothelial carcinoma and non-neoplastic changes shows extensive overlap, making a reliable diagnosis impossible. J Am Soc Cytopathol 4:90–97 31051715 10.1016/j.jasc.2014.10.006

[6] Mcintire PJ, Aragao A, Burns BL et al (2022) Digital image analysis of high-grade urothelial carcinoma in urine cytology confirms chromasia heterogeneity and reveals a subset with hypochromatic nuclei and another with extremely dark or “India ink” nuclei. Cancer 130:363–369 10.1002/cncy.22554 35104393

[7] Mcintire PJ, Elsoukkary SS, Robinson BD et al (2021) High-grade urothelial carcinoma in urine cytology: different spaces—different faces, highlighting morphologic variance. J Am Soc Cytopathol 10:36–40 32958411 10.1016/j.jasc.2020.08.001

[8] Owens CL, Ali SZ (2005) Atypical squamous cells in exfoliative urinary cytology: clinicopathologic correlates. Diagn Cytopathol 33:394–398 16299739 10.1002/dc.20344

[9] Pierconti F, Martini M, Straccia P et al (2018) Hypochromatic large urothelial cells in urine cytology are indicative of high grade urothelial carcinoma. APMIS 126:705–709 30160022 10.1111/apm.12877

[10] Rosenthal DL, Wojcik EM, Kurtycz DF (2016) The Paris system for reporting urinary cytology. Springer, Berlin 10.1016/j.jasc.2021.12.003 35094954

[11] Roupret M, Seisen T, Birtle AJ et al (2023) European Association of Urology Guidelines on Upper Urinary Tract Urothelial Carcinoma: 2023 Update. Eur Urol 84:49–64 36967359 10.1016/j.eururo.2023.03.013

[12] Sassa N, Iwata H, Kato M et al (2019) Diagnostic Utility of UroVysion Combined With Conventional Urinary Cytology for Urothelial Carcinoma of the Upper Urinary Tract. Am J Clin Pathol 151:469–478 30668617 10.1093/ajcp/aqy170

[13] Wojcik EM, Kurtycz DF, Rosenthal DL (2022) The Paris System for Reporting Urinary Cytology. Springer, Cham 10.1016/j.jasc.2021.12.003 35094954

[14] Wojcik EM, Kurtycz DFI, Rosenthal DL (2022) We’ll always have Paris. The Paris System for Reporting Urinary Cytology 2022. J Am Soc Cytopathol 11:62–66 35094954 10.1016/j.jasc.2021.12.003

[15] Zhang ML, Rosenthal DL, Vandenbussche CJ (2016) The cytomorphological features of low-grade urothelial neoplasms vary by specimen type. Cancer 124:552–564 10.1002/cncy.21716 27019161

